# Green Preparation of Fluorescent Nitrogen-Doped Carbon Quantum Dots for Sensitive Detection of Oxytetracycline in Environmental Samples

**DOI:** 10.3390/nano10081561

**Published:** 2020-08-08

**Authors:** Rong Gao, Zhibin Wu, Li Wang, Jiao Liu, Yijun Deng, Zhihua Xiao, Jun Fang, Yunshan Liang

**Affiliations:** 1 Hunan Engineering Laboratory for Pollution Control and Waste Utilization in Swine Production, College of Bioscience and Biotechnology, Hunan Agricultural University, Changsha 410128, China; gaorong0130@163.com (R.G.); wzbaaa11@hunau.edu.cn (Z.W.); 2 Hunan Provincial Key Laboratory of Rural Ecosystem Health in Dongting Lake Area, College of Resources and Environment, Hunan Agricultural University, Changsha 410128, China; wliiris1024@163.com (L.W.); liujiao913@163.com (J.L.); dengyijun9910@163.com (Y.D.); xiaozhihua@hunau.edu.cn (Z.X.)

**Keywords:** nitrogen-doped carbon quantum dots, natural biomass, fluorescence, oxytetracycline, detection

## Abstract

Nitrogen-doped carbon quantum dots (N-CQDs) with strong fluorescence were prepared by a one-step hydrothermal method using natural biomass waste. Two efficient fluorescent probes were constructed for selective and sensitive detection of oxytetracycline (OTC). The synthesized N-CQDs were characterized by UV-visible absorption spectra, fluorescence spectra, Fourier transform infrared spectroscopy (FT-IR), X-ray photon spectroscopy (XPS), atomic force microscopy (AFM), and high-resolution transmission electron microscopy (HRTEM), which proved that the synthesized N-CQDs surface were functionalized and had stable fluorescence performance. The basis of N-CQDs detection of OTC was discussed, and various reaction conditions were studied. Under optimized conditions, orange peel carbon quantum dots (ON-CQDs) and watermelon peel carbon quantum dots (WN-CQDs) have a good linear relationship with OTC concentrations in the range of 2–100 µmol L^−1^ and 0.25–100 µmol L^−1^, respectively. ON-CQDs and WN-CQDs were both successfully applied in detecting the OTC in pretreated tap water, lake water, and soil, with the recovery rate at 91.724–103.206%, and the relative standard deviation was less than 5.35%. The results showed that the proposed N-CQDs proved to be green and simple, greatly reducing the detection time for OTC in the determination environment.

## 1. Introduction

Antibiotics are natural, synthetic, or semisynthetic compounds that interfere with the development of other living cells [1]. The global consumption of antibiotics continues to increase, mainly due to the increase in the use of drugs and the demand for animal protein due to the increase in population [1,2]. Oxytetracycline (OTC) is a popular growth accelerator for livestock and aquaculture because of its low cost and strong antibacterial activity [3,4], while it can stay in edatope for more than one year, which is longer than other types of antibiotics [5]. Previous studies have shown that the OTC median in raw sludge was higher than 10 µg kg^−1^ dry matter [6], and the highest TC concentration detected in agricultural soils was 0.6 mg kg^−1^ [7,8]. While the OTC in Honghu, China, had a maximum concentration of 2796.6 ng L^−1^ [9]. In addition, the comparisons in animal excrement data showed that OTC was consistently the highest average compound [10]. Even more worryingly, antibiotic resistance genes caused by overuse of antibiotics can be transferred between environmental bacteria and human pathogens [11]. While the soil is an important reservoir of antibiotic resistance genes in the environment, resistance genes can be transferred to groundwater or lakes by leaching [12,13]. The amount of antibiotics discharged from pharmaceutical factories, hospitals, and farms in the environment has reached mg L^−1^ level [2,14]. Therefore, it was very necessary to detect antibiotics in the environment, which can effectively identify the residual concentration of antibiotics, improve the detection efficiency, and shorten the detection time, thus indirectly accelerating the pollution prevention and control process.

To date, approaches for detection of OTC mainly include liquid chromatography-tandem mass spectrometry (LC-MS/MS) [15], semiquantitative lateral flow assay (LFA) [16], chemiluminescence [17], as well as enzymatic catalysis [18]. These methods have high stability and sensitivity; however, their applications remain limited due to the complex requirements of expensive equipment and experimental operations [19]. Actually, fluorescence analysis has the advantages of high sensitivity, low cost, and simple operation, which has aroused great interest in the detection field [20,21,22]. Moreover, the fluorescence method can be applied to the detection of various organic pollutants and metal ions in the environment, including antibiotics (OTC [3], tetracycline [23,24], chlortetracycline [24], enrofloxacin [25]), metal ions (Au^3+^ [26], Hg^2+^ [27,28], Fe^3+^ [24,29,30]), pesticides [31,32] 2,4,6 trinitrophenol [33,34], glutathione [35,36], and chitosan [37].

Carbon quantum dots (CQDs) are a kind of nano-sized spherical carbon fluorescent material with unique advantages of light stability and sensitivity [30,38,39]. So far, different types of biomass materials, including lycium [40], hair [27], sugarcane bagasse pulp [41], Citrus limon peels [42], orange juice [28], tobacco [43], watermelon juice [30], corn juice [44], and carica papaya juice [45] have been used to synthesize CQD. Biowaste was seen as a potential substitute for certain chemicals and waste peels contain carbohydrates, such as fructose, glucose, sucrose, and cellulose [46]. Chatzimitakos et al. synthesized two kinds of CQDs with similar properties from Citrus sinensis and Citrus limon peels using a carbonization process. The results showed that the correlation detection performance of the two kinds of CQDs synthesized from different peels were different, which may be because of their unique analytical properties, which supported their differences [42]. Furthermore, several nitrogenous materials, including rice residue and xylan, have been used to prepare N-CQD to detect antibiotics [23,24]. Using rice residue and glycine as carbon and nitrogen sources, Qi et al. [24] synthesized N-CQD with high quantum yield through one-step hydrothermal method, and this N-CQD was used as a fluorescent probe for the detection of Fe^3+^, tetracycline, OTC, and aureomycin in actual water samples and cell imaging. The results of the above studies indicated that N-CQD showed excellent solubility, sensitive selectivity, high stability, and good biocompatibility. However, the reaction time of nitrogen doping up to 12 h was more time-consuming and complex, which was not conducive to experimental operation. Yang et al. [23] could synthesize N-CQD with xylan ammonia solution as the precursor in only 10 min, which had excellent photoluminescence properties and a high tolerance to salt and metal ions. Due to the internal filter effect, N-CQD showed high selectivity and sensitivity to tetracycline antibiotics. However, the extraction of xylan was more complex than using common biomass materials, and the purity and stability requirements were higher. The xylan in Yang et al. was purchased from the company. It was proved that the direct preparation of N-CQD by hydrothermal method was an effective method to improve the quality of CQD [47,48]. Heteroatomic doping of CQD not only improves fluorescence efficiency but also provides active sites in CQD to expand its potential applications in analysis and sensing [24]. Through the synergistic coupling effect, heteroatom-doped carbon materials can produce unique electronic structures with higher active sites [49,50]. In particular, nitrogen doping plays an important role in the regulation of electronic and chemical properties of carbon materials. Carbon atoms are replaced by nitrogen to produce posture and induce *n*-type conductivity [51,52]. Some studies have shown that the density and bonding configuration of dopants can change the physical properties, such as electron and magnetic properties [52].

In this study, waste orange peel and watermelon peel with high carbohydrate content were used as a green carbon source for N-CQDs preparation via a one-step hydrothermal method in ammonia solution. This was a simple green chemical method for preparing large-scale fluorescent carbon dots. The N-CQDs were characterized systematically and the basic conditions for detection of OTC were discussed. Ultimately, synthetic N-CQDs were used for OTC detection in tap water, lake water, and soil treatment samples.

## 2. Experimental

### 2.1. Reagents and Chemicals

The OTC standard (95%) was purchased from Aikeda chemical reagent Co. Ltd. (Chengdu, China). The chemical structure of OTC is shown in Figure S1. Ammonia, phosphoric acid, boric acid, acetic acid, sodium hydroxide (NaOH), and anhydrous ethanol were purchased from Sinopharm Chemical Reagent Co. Ltd. (Shanghai, China). All chemicals used were of analytical reagent grade. Britton-Robinson (BR)buffer solution was prepared by a 0.04 mol L^−1^ solution of boric acid, phosphoric acid, and acetic acid (mixed at a ratio of 1:1:1), and the pH value was adjusted to 2–12 with 0.2 mol L^−1^ NaOH solution. The experiment was produced using ultrapure water (Kertone, Changsha, China) with an initial resistivity of 18.25 MΩ.

### 2.2. Preparation of N-CQDs

Oranges (navel oranges) and watermelons (desert melons in Ningxia) were purchased from fruit stores. The fresh peel (orange peel and watermelon peel) were cut into pieces and dried at 80 °C until the water was completely removed. Then, the sample was broken with a crusher, and the fine particles were selected by using a standard screen (400 mesh) 3 times. We added 0.03 g of orange peel or watermelon peel powder to the lined beaker, then 3 mL of ammonia water and 17 mL of deionized water in this order. After mixing and stirring them, they were put into a tetrafluoroethylene liner beaker and then transferred to a reaction kettle. Orange peel powder was heated at 100 °C for 5 h, and watermelon peel powder was heated at 140 °C for 2 h. After cooling to room temperature, pale yellow solutions were obtained. After centrifugation (10,000 rpm, 10 min), the supernatant was taken and the large particles were removed by 0.22 µm ultrafiltration. The dialysis was then performed under a 500-molecular-weight dialysis bag for two days with the water changed every 2 h. From this, the reserve orange peel nitrogen carbon doped quantum (ON-CQDs) and watermelon peel nitrogen carbon doped quantum dots (WN-CQDs) were obtained.

### 2.3. Characterization of N-CQDs

The morphologies and sizes of N-CQDs were characterized by transmission electron microscopy (TEM, Tecnai G2 F20, FEI, Hillsborough, Oregon, USA) and multimode V atomic force microscopy (AFM, Veeco, Plainview, NY, USA). TEM and high-resolution transmission electron microscopy (HRTEM, FEI, Hillsborough, Oregon, USA) was scanned at 200 kV. The aqueous solution of the N-CQDs sample was dropped on a 300 mesh ultra-thin carbon coated copper mesh, and then the copper mesh was dried in a vacuum drying box for measurement. The obtained TEM images were analyzed and processed using Gatan Digital Micrograph software. The multifunctional imaging electron spectrometer (XPS, Thermo, Waltham, MA, USA) was collected using a monochromatic Al KaXray source (hv = 1486.6 eV) with a power of 150 W and a beam spot of 500 µm, and its binding energy was calibrated at C1s 284.8. The measurement range of FTIR spectrum was 500–4000 cm^−1^. The sample liquid was ground together with potassium bromide and measured after being tableted and dried. For F-7000 fluorescence spectrometric measurements (Hitachi High Technologies, Tokyo, Japan), excitation and emission slits were set at 5 nm, and the excitation light-source wavelengths were set at 320 nm (ON-CQDs) and 280 nm (WN-CQDs). The fluorescence quantum yield of N-CQDs was determined by absolute method (also known as absolute quantum yield) [53,54,55]. Time-resolved fluorescence decay curve measurements were also performed on the FLS980 series of fluorescence spectrometers (Edinburgh Instruments, Livingston, UK).

### 2.4. Pretreatment and Preparation of Environmental Samples

Samples of tap water, lake water, and soil were taken from the university campus. Tap water samples were obtained in the laboratory, and lake water samples were collected in front of the laboratory building. The water sample was first settled naturally for 24 h, and then the suspended particles in the lake were filtered with a filter paper with a maximum aperture of 15–20 μm. The filtered water samples were centrifuged at 10,000 rpm for 20 min, and the supernatant was collected and stored in a refrigerator at 4 °C for later use. Soil sampling was carried out by removing the surface soil about 1–2 cm with a scraper, and soil samples were collected rapidly in the new soil section. The samples were naturally air-dried for 48 h and then dried in an oven at 100 °C. The fine particles were selected by using a standard screen (400 mesh) 3 times, and then the dried soil samples were crushed and weighed accurately 1 g. The samples were placed in a hermetically sealed centrifuge tube, followed by mechanical oscillation of 4 mL acetonitrile for 30 min, centrifugation with 5000 r min^−1^ for 10 min, and the supernatant was collected and stored in the centrifuge tube at 4 °C. All standby samples were filtered using a 0.22 μm microporous membrane.

## 3. Results and Discussion

### 3.1. Morphologies of N-CQDs

**Figure 1:**
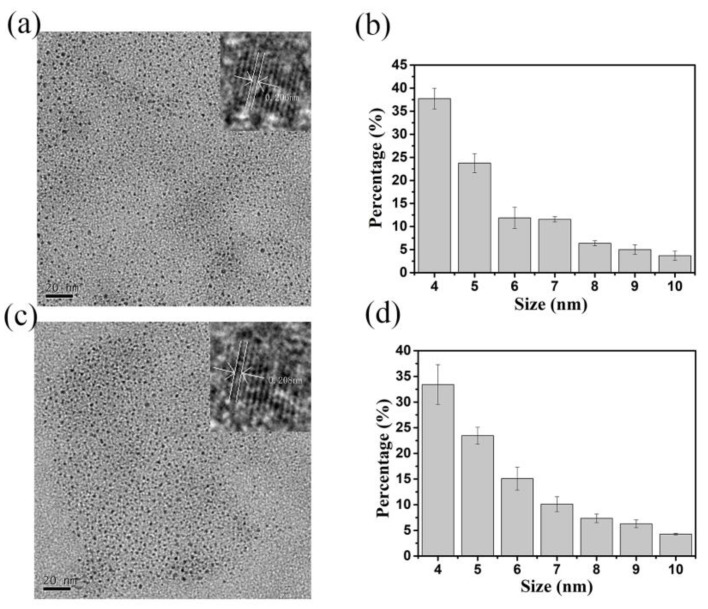
Transmission electron microscopy (TEM) image of the nitrogen-doped carbon quantum dots (N-CQDs). (**a**) orange peel nitrogen carbon doped quantum (ON-CQDs), (**c**) watermelon peel carbon quantum dots (WN-CQDs); (inset: high-resolution transmission electron microscopy (HRTEM) of the N-CQDs); (**b**) and (**d**) shows the size distribution of the carbon dots corresponding to (**a**) and (**c**), respectively.

**Figure 2:**
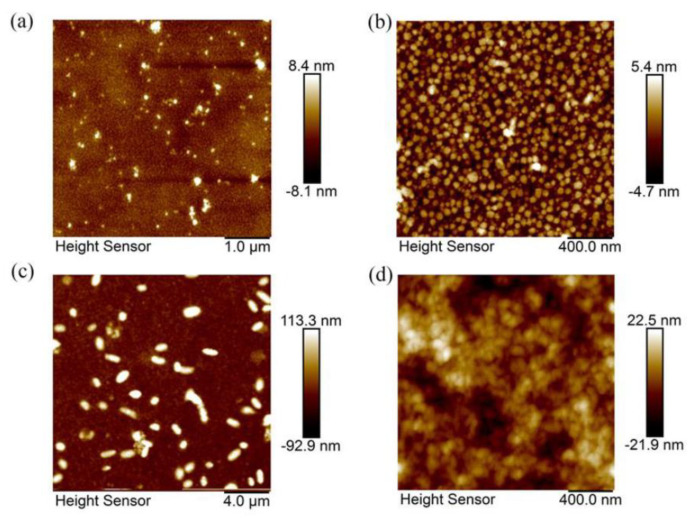
Atomic force microscope (AFM) images of N-CQDs. Low-magnification images of N-CQDs: (**a**) ON-CQDs (5 µm) and (**c**) WN-CQDs (20 µm). (**b**,**d**) are high-magnification images (2 µm) of ON-CQDs and WN-CQDs, respectively.

The size and morphology of the synthesized CQDs were analyzed by transmission electron microscopy (TEM) and high-resolution transmission electron microscopy (HRTEM). TEM results showed that both types of N-CQDs had good uniformity and dispersed, and the average particle size was 5 ± 0.5 nm. HRTEM images of ON-CQDs and WN-CQDs were shown in Figure 1a and c, respectively, indicating that ON-CQDs lattice spacing was 0.206 nm and WN-CQDs lattice spacing was 0.208 nm, which was consistent with the (100) plane spacing of sp^2^ carbon [56,57,58]. The particle size distribution diagrams were shown in Figure 1b and d. It can be found that the microscopic morphology of the two N-CQDs were similar. The particle size distribution of both N-CQDs range from 4 to 10 nm, with the maximum distribution at 4 nm. Figure 2 shows the low-magnification images were 5, 20 µm (Figure 2 a and c) and high-magnification images were 2 µm (Figure 2 b and d) of 2D AFM of N-CQDs, which were selected according to the sample morphology [41,59]. It can be seen that acceptable N-CQDs’ distribution was obtained in terms of size and shape. Consistent with the TEM results, it was observed that N-CQDs were small, spherical, and the surface roughness was less than 5 nm.

### 3.2. Composition of N-CQDs

**Figure 3:**
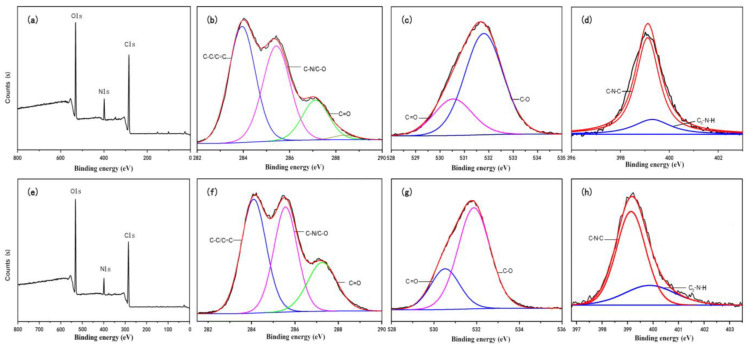
XPS characterizations of N-CQDs. Full spectrum: (**a**,**e**); Cls: (**b**,**f**); Ols: (**c**,**g**); Nls: (**d**,**h**), represents ON-CQDs and WN-CQDs, respectively.

XPS analysis shows the elemental composition, chemical state, and molecular structure of N-CQDs. The XPS survey spectrum showed that the peaks were 284.79, 399.02, and 531.10 eV in Figure 3a (ON-CQDs) and 285.05, 399.12, and 531.59 eV in Figure 3e (WN-CQDs), corresponding to C1s, N1s, and O1s electrons, respectively. The high-resolution spectrum of the C1s signal shows three peaks at 283.97, 285.42, 287.12 eV (Figure 3b) and 284.10, 285.57, 287.26 eV (Figure 3f), representing the C-C/C=C, C-N/C-O, and C=O groups, respectively [60]. The O1s spectrum (Figure 3c and g) shows two fitting peaks at 530.57, 531.82 eV, and 530.52, 531.87 eV, belonging to C=O and C-O groups, respectively. The high-resolution spectrum of N1s shows two peaks at 399.11, 399.3 eV (Figure 3d) and 399.14, 399.87 eV (Figure 3h), which can be designated as C-N-C and C_2_-N-H functional groups, respectively, indicating successful nitrogen doping into CQDs [61,62].

**Figure 4:**
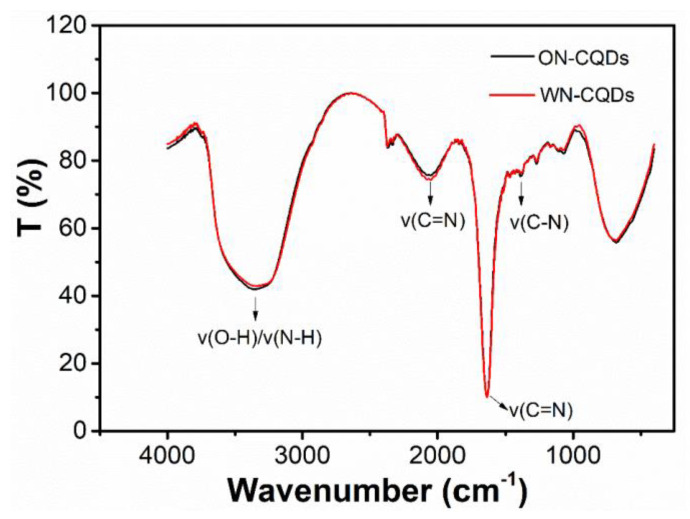
FTIR spectrum of ON-CQDs (black) and WN-CQDs (red).

In addition, FTIR spectra were employed to confirm the existence of functional groups. As shown in Figure 4, the peaks at 3354 cm^−1^ and 3339 cm^−1^ could be attributed to the characteristic absorption of O-H by tensile vibration, while the peaks of C=O and C=N are at 1637 cm^−1^ and 2075 cm^−1^, 2048 cm^−1^. Meanwhile, the peaks at 1250 cm^−1^ were attributed to the C–N out-of-plane bending vibration [57]. Furthermore, the N-CQDs produced here contain characteristic functional groups, such as -COOH, -OH, and -C-N-C, and all of the data show that it was consistent with XPS [43].

### 3.3. Optical Properties of N-CQDs

**Figure 5:**
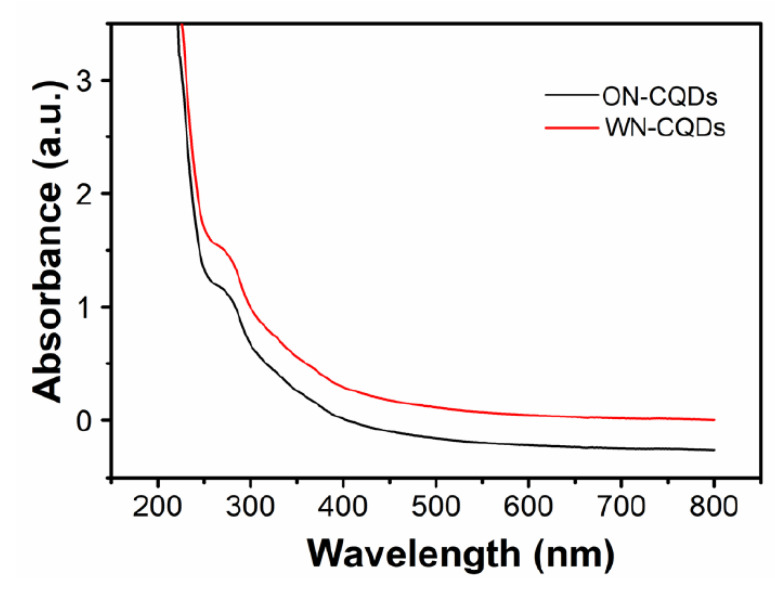
UV-Vis spectrum of ON-CQDs (black) and WN-CQDs (red).

**Figure 6:**
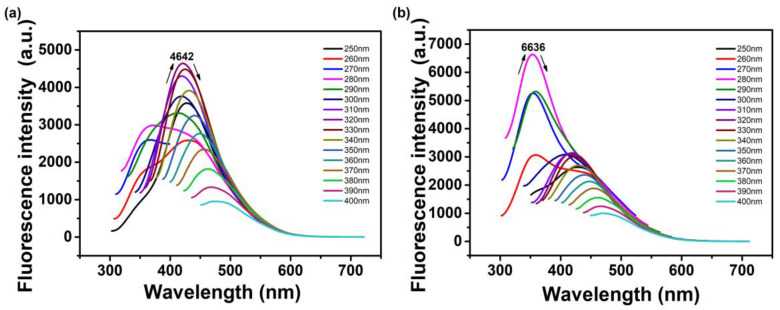
Excitation-dependent fluorescence emission spectra of N-CQDs: (**a**) ON-CQDs; (**b**) WN-CQDs.

The optical properties of the prepared N-CQDs were analyzed by UV-visible spectroscopy and fluorescence spectrum. The samples were diluted to 0.2 ± 0.05 mg/mL with deionized water. In the UV−vis absorption spectrum (Figure 5), ON-CQDs and WN-CQDs presented two obvious absorption peaks at around 265 and 280 nm, respectively. The peaks at 265 and 280 nm were ascribable to the π–π* transition for the C=C or C=O bond in which the orbital was sp^2^ hybridized clusters [24,26,27,63]. The fluorescence emission spectra of N-CQDs under different excitations were shown in Figure 6. When the wavelength of the excitation light increased from 250 to 400 nm, the emission peaks were all redshifted. In particular, the excitation wavelength of ON-CQDs produces a corresponding blue shift in the peak position of the emission spectrum at 270 nm and 280 nm. This may be due to the increase in the band gap, and the band gap transition can regulate the fluorescence variation of ON-CQDs [43]. Studies have shown that the nitrogen-doping process can lead to significant redshift of the optimal excitation wavelength and the strongest emission peak, which may depend on the different sizes of distribution of N-CQDs and the characteristics of multi-surface emission points. [26,29,64]. The maximum excitation wavelengths (λ _ex_) of ON-CQDs (Figure 6a) and WN-CQDs (Figure 6b) were 320 nm and 280 nm, respectively. Studies have also shown that excitation-related emission behaviors may be attributed to the presence of different functional groups to determine the degree and nature of aggregation [44,65].

**Figure 7:**
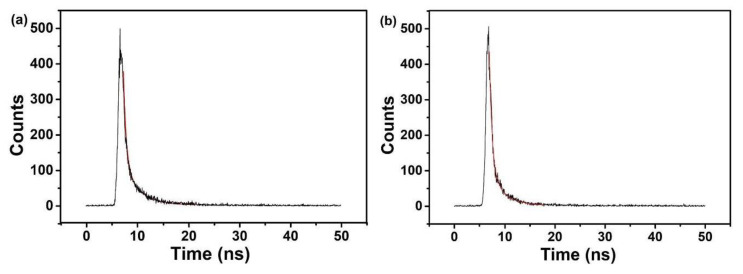
Fluorescence attenuation curve and fitting results: (**a**) ON-CQDs; (**b**) WN-CQDs.

The time-resolved fluorescence decay curve, as shown in Figure 7, uses the formula ($f\left(t\right)=a_{1}e^{-t/\tau_{1}}+a_{2}e^{-t/\tau_{2\;}}\;with\;a_{1}+a_{2}=1$), fitting to a double-exponential decay model function, where τ_1_, τ_2_ are the fast and slow lifetime components, and a_1_, a_2_ are the corresponding amplitudes [66]. The change of fluorescence intensity of N-CQDs was a gradual attenuation process, and there were two fluorescent groups in N-CQDs, respectively. The two fluorescence lifetimes indicate the existence of different luminous states, which indicates that the surface morphology of N-CQDs were not uniform, and there were multiple luminous structures, showing the characteristics of excitation dependence and multiple fluorescence lifetimes [67]. The fluorescence lifetimes of the two fluorophores in ON-CQDs (Figure 7a) were 0.54 ns and 2.95 ns, and the relative concentrations were 29.78% and 70.22%, respectively; the fluorescence lifetimes of the two fluorophores in WN-CQDs (Figure 7b) were 0.56 ns and 2.79 ns, and the relative concentrations were 43.19% and 56.81%, respectively. According to literature, the shorter lifetime comes from surface-related recombination, which was easily affected by the size of N-CQDs and its surface chemistry [68,69]. The fitting results of N-CQDs were comprehensively observed, and it was found that both of them contained a fluorescence lifetime around 0.5 ns and 2.8 ns. It can be inferred that both types of N-CQDs have fluorophores corresponding to this lifetime or different structures of the same fluorescent substance, and the fluorophores corresponding to 2.95 ns and 2.79 ns with a long fluorescence life account for a relatively large contribution rate in the whole decay process. The absolute quantum yields of ON-CQDS and WN-CQDS were calculated as 6.86% and 7.53%, respectively.

### 3.4. Basis of OTC Detection

In order to obtain the best detection performance, several key factors affecting the sensitivity and stability of the experiment were studied, such as the pH value of the BR buffer solution (Figure S2), the volume of the BR buffer solution (Figure S3a,b), the order of reagent addition (Figure S4a,b), and the different reaction times (Figure S5). The results showed the best quenching effect on ON-CQDs when the concentration of OTC was 40 µmol L^−1^, the order of addition was OTC+BR + ON-CQDs, the BR buffer was 1 mL with a pH value of 10, and the incubation time was 20 min. The quenching effect was best when the concentration of OTC was 80 µmol L^−1^, and the BR buffer was 3 mL with a pH value of 8. The order of adding was OTC + WN-CQDs + BR, which basically stabilized at 20 min.

**Figure 8:**
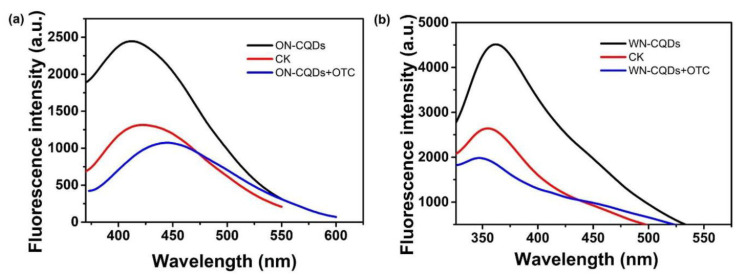
Fluorescence emission spectra of N-CQDs, CK (N-CQDs + deionized water), N-CQDs + OTC. (**a**) ON-CQDs (λ _ex_ = 320 nm); (**b**) WN-CQDs (λ _ex_ = 280 nm).

To investigate the fluorescence quenching of N-CQDs, OTC was added to N-CQDs to form an N-CQDs + OTC composite system. After 20 min, the emission spectra of N-CQD, N-CQDs + OTC, and CK (N-CQDs + deionized water) were measured, respectively. As shown in Figure 8a (ON-CQDs) and b (WN-CQDs), the fluorescence intensity of N-CQDs decreased slightly after the addition of deionized water, which was due to the dilution of the N-CQDs solution. OTC was added to the N-CQDs solution to form the N-CQDs + OTC system, and the fluorescence of N-CQDs fluorescence significantly decreased by about 50%. This was due to the presence of -NH_2_ in N-CQDs, which has the fluorescence resonance energy transfer with electron-deficient aromatic rings in the OTC structure, which was consistent with the mechanism reported in the literature [70]. Additionally, the good selectivity results of ON-CQDs (Figure S6a) and WN-CQDs (Figure S6b) were shown in Figure S6, respectively.

### 3.5. Linear Range and Detection Limit of OTC

**Figure 9:**
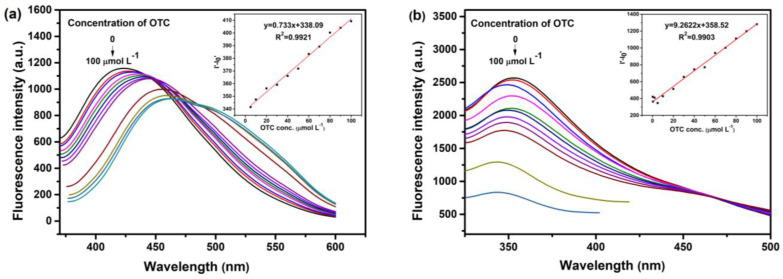
Emission spectra of aqueous N-CQDs dispersion upon addition of various concentrations of OTC. (**a**) ON-CQDs (from top to bottom: 0, 2, 5, 10, 20, 30, 40, 50, 60, 70, 80, 90, and 100 µmol L^−1^); (**b**) WN-CQDs (from top to bottom: 0, 0.25, 0.5, 2, 5, 10, 20, 30, 40, 50, 60, 70, 80, 90, and 100 µmol L^−1^). Inset: calibration curve of fluorescence intensity difference with OTC concentration.

The sensitivity of N-CQDs to OTC measurement was measured under optimized experimental conditions (Figure 9). Fluorescence intensity difference (ΔF = I′ − I′_0_) decreases with the increase in OTC concentration, where I′ and I′_0_ were, respectively, the fluorescence intensity of N-CQDs in the absence and presence of OTC. ON-CQDs (Figure 9a) and WN-CQDs (Figure 9b), respectively, an OTC concentration of 2–100 µmol L^−1^ and 0.25–100 µmol L^−1^ showed a good linear relationship within the scope of the linear regression equation, respectively, ΔF = 0.733 C(µmol L^−1^) + 338.09 and ΔF = 9.2622 C (µmol L^−1^) + 358.52, and the linear correlation coefficient (R^2^) were 0.9921 and 0.9903, respectively [71,72]. Furthermore, the detection limit was calculated to be 0.973 µmol L^−1^ (ON-CQDs) and 0.077 µmol L^−1^(WN-CQDs) based on 3σ/k, where σ and k represent the standard deviation and slope of the curve, respectively. The difference in the detection limit may be caused by the different composition of two N-CQD raw materials. In addition, the analytical performance of this work was compared with the previous detection methods of OTC (Table 1). The results showed that the detection limit of this experiment was lower than most of the reported methods, the linear range was wide, and no complex chemical modification was required.

### 3.6. Determination of OTC in Environmental Samples

Under experimentally optimized conditions, different concentrations of OTC standard solutions were added to pre-treated tap water, lake water, and soil samples and then measured according to the experimental method. Each concentration was measured in parallel five times. As can be seen from Table 2, the recovery of OTC in tap water, lake water, and soil samples were 91.724–103.206%, and the relative standard deviation (RSD) was less than 5.35%. In Table S1, the results were similar and satisfactory to the recoveries of OTC in other samples. It shows that this work can be applied to the detection of antibiotic organic pollutants in the environment.

## 4. Conclusions

In conclusion, two kinds of N-CQDS were synthesized by a hydrothermal method using waste natural biomass materials (orange peel and watermelon peel) without pre/post chemical treatment. The average particle size at 5 nm of the N-CQDs were both small, both of them contained -COOH, -OH, -NH_2_, -C-N-C groups, and the fluorescence spectrum was red-shifted as the excitation wavelengths increased. Then, based on the fluorescence quenching responses of N-CQDs, fluorescence probes for OTC detection were successfully constructed with good sensitivity, and detection limits under the optimal reaction conditions were 0.973 µmol L^−1^ (ON-CQDs) and 0.077 µmol L^−1^ (WN-CQDs), respectively. This work was not only environmentally friendly and avoided complex synthetic steps but can also be successfully applied to OTC detection and analysis in the real environment, and it also showed great potential for analyzing trace organic matter in the natural environment.

## Figures and Tables

**Table 1:** Comparison with previous methods of OTC analysis.

Method	Linear Range (µmol L^−1^)	Detection Limit (µmol L^−1^)	Citations
N-CQDs fluorescent probe	3.32–32.26	0.3739	[24]
Fluorescent sensor for OTC based on SiNPs	0.2–20	0.18	[4]
Gold nanoclusters fluorescent probe	0.375–12.5	0.15	[73]
BODIPY fluorescent probe	0–42	0.72	[74]
ON-CQDs fluorescent probe	2–100	0.973	This work
WN-CQDs fluorescent probe	0.25–100	0.077	This work

**Table 2:** Determination of OTC in environmental samples.

Samples	Spiked	Measured Concentration	Recovery Rate (%)	RSD (%, *n* = 5)
Lake water ^1^	5 µmol L^−1^	4.870 µmol L^−1^	97.408	0.62%
Tap water ^1^	10 µmol L^−1^	9.754 µmol L^−1^	97.544	0.94%
Soil ^1^	12.240 µmol Kg^−1^	12.632 µmol Kg^−1^	103.206	0.82%
Tap water ^1^	30 µmol L^−1^	27.517 µmol L^−1^	91.724	1.06%
Lake water ^1^	40 µmol L^−1^	40.341 µmol L^−1^	100.853	0.62%
Soil ^1^	30.600 µmol Kg^−1^	31.468 µmol Kg^−1^	102.837	0.88%
Tap water ^2^	2 µmol L^−1^	1.844µmol L^−1^	92.203	0.84%
Soil ^2^	9.180 µmol Kg^−1^	9.255 µmol Kg^−1^	100.826	0.81%
Lake water ^2^	20 µmol L^−1^	19.399µmol L^−1^	96.996	1.05%
Tap water ^2^	35 µmol L^−1^	34.514µmol L^−1^	98.613	0.94%
Lake water ^2^	45 µmol L^−1^	45.311µmol L^−1^	100.691	0.88%
Soil ^2^	30.600 µmol Kg^−1^	31.469 µmol Kg^−1^	102.844	5.35%

Note: sample ^1^ were ON-CQDs; sample ^2^ were WN-CQDs.

## Supplementary Materials

The following are available online at https://www.mdpi.com/2079-4991/10/8/1561/s1, Figure S1: The chemical structure of OTC, Figure S2: Fluorescence performance of ON-CQDs (black) and WN-CQDs (red) at various pH values, Figure S3: Effect of buffer solution volume on fluorescence intensity of N-CQDs: (a) ON-CQDs; (b) WN-CQDs, Figure S4: Effect of reagent adding sequence on fluorescence intensity of N-CQDs: (a) ON-CQDs; (b) WN-CQDs, Figure S5: Fluorescence performance of ON-CQDs (black) and WN-CQDs (red) at different reaction times, Figure S6: Selectivity of N-CQDs: (a) ON-CQDs; (b): WN-CQDs. Concentrations of various substances: (a), (b) correspond to 40µmol L^−1^ and 80µmol L^−1^ respectively. (I′ and I′_0_ represent the fluorescence intensity of N-CQDs in the presence and absence of various substances, respectively). Table S1: Compare OTC recovery rates in various samples.
