# Supplementary material for: Green Preparation of Fluorescent Nitrogen-Doped Carbon Quantum Dots for Sensitive Detection of Oxytetracycline in Environmental Samples
Source: Nanomaterials (Basel). 2020 Aug 8;10(8):1561. doi: 10.3390/nano10081561 (PMC7466531; doi:10.3390/nano10081561)
Supplement: Supplementary file 1 [file nanomaterials-10-01561-s001.pdf]

nanomaterials

Supplementary Materials

Green Preparation of Fluorescent Nitrogen-Doped Carbon Quantum Dots for Sensitive Detection of Oxytetracycline in Environmental Samples

Rong Gao [1,2,†], Zhibin Wu [1,2,†], Li Wang [2], Jiao Liu [2], Yijun Deng [2], Zhihua Xiao [2], Jun Fang [1,2,*] and Yunshan Liang [1,2,*]

[1] Hunan Engineering Laboratory for Pollution Control and Waste Utilization in Swine Production, College of Bioscience and Biotechnology, Hunan Agricultural University, Changsha 410128, China; gaorong0130@163.com (R.G.); wzbaaa11@hunau.edu.cn (Z.W.)
[2] Hunan Provincial Key Laboratory of Rural Ecosystem Health in Dongting Lake Area, College of Resources and Environment, Hunan Agricultural University, Changsha 410128, China; wliiris1024@163.com (L.W.); liujiao913@163.com (J.L.); dengyijun9910@163.com (Y.D.); xiaozhihua@hunau.edu.cn (Z.X.)
* Correspondence: fangjun1973@hunau.edu.cn (J.F.); lyss3399@126.com (Y.L.); Tel.: +86-731-8461-3600 (J.F.)
† These authors contributed equally to this work.

Selectivity

We verified the good selectivity of the two fluorescent probes with three similar structural tetracycline antibiotics (tetracycline, doxycycline, and oxytetracycline) [1,2]. Fluorescence quenching reaction between N-CQDs and metal ions and organics in several environments was observed. Figure S6a showed that the fluorescence intensity of ON-CQDs was not greatly affected, while Figure S6b showed that the fluorescence quenching of WN-CQDs occurred. In contrast, oxytetracycline (OTC) addition resulted in a significant quenching of the original fluorescence intensity. Tetracycline and doxycycline showed small changes compared with OTC. This indicates that among these metal ions and antibiotics, N-CQDS has a strong absorbance spectral response to OTC [2].

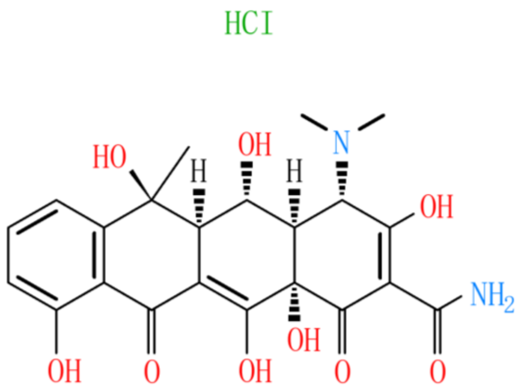

Figure S1. The chemical structure of OTC.

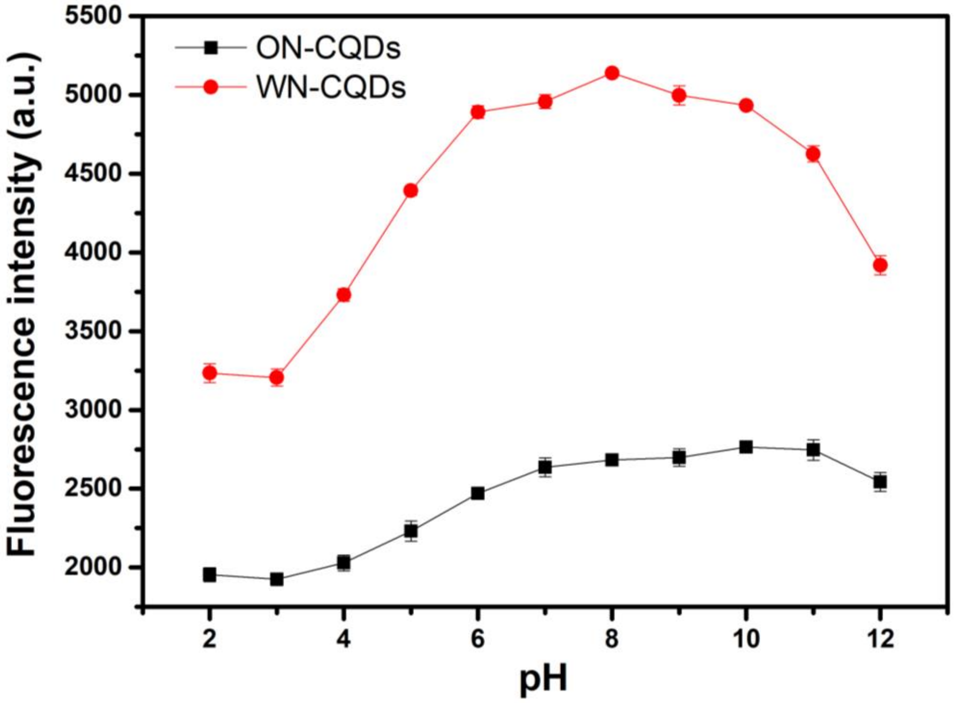

Figure S2. Fluorescence performance of ON-CQDs (black) and WN-CQDs (red) at various pH values.

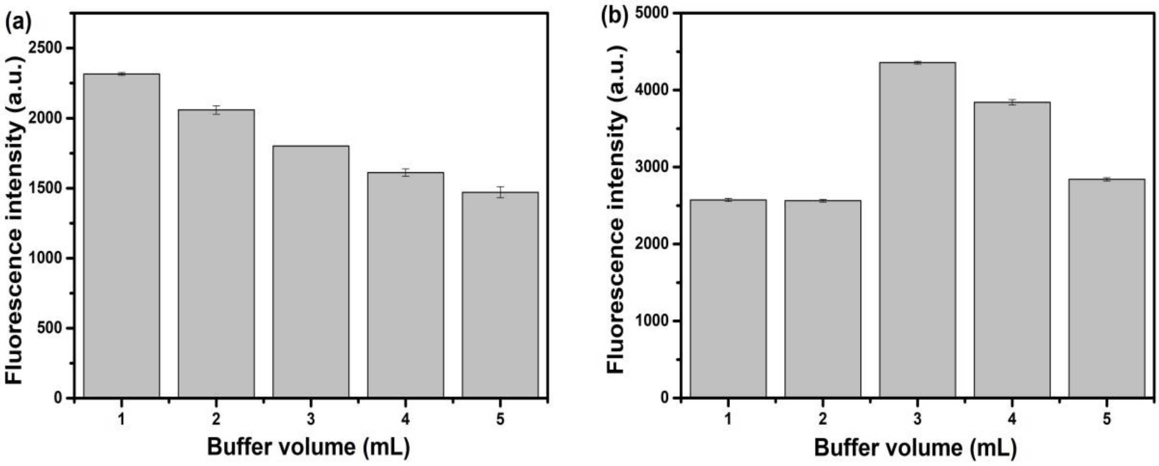

Figure S3. Effect of buffer solution volume on fluorescence intensity of N-CQDs: (**a**) ON-CQDs; (**b**) WN-CQDs.

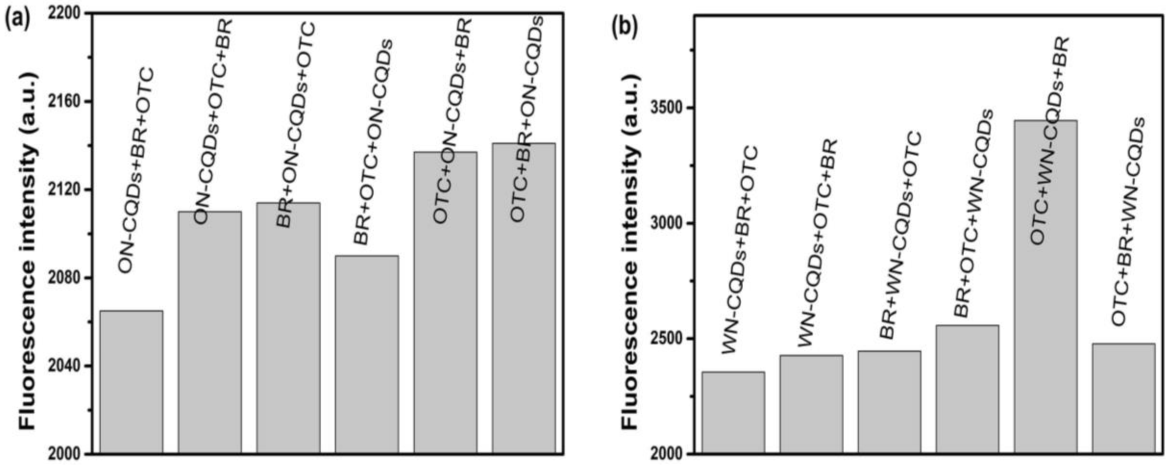

Figure S4. Effect of reagent adding sequence on fluorescence intensity of N-CQDs: (**a**) ON-CQDs; (**b**) WN-CQDs.

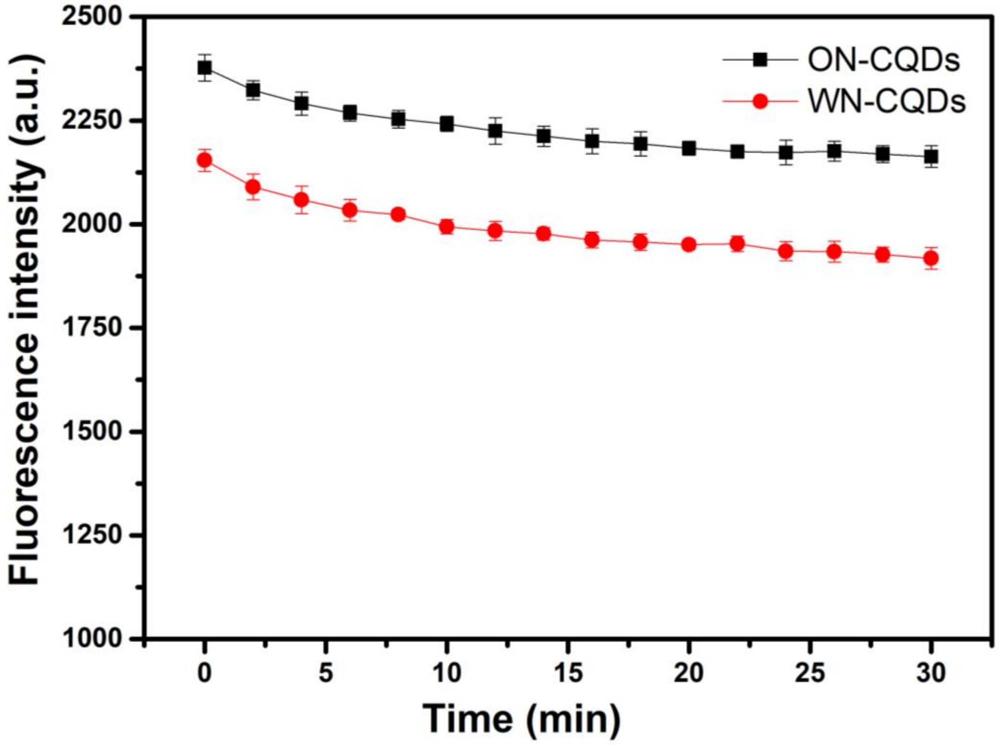

Figure S5. Fluorescence performance of ON-CQDs (black) and WN-CQDs (red) at different reaction times.

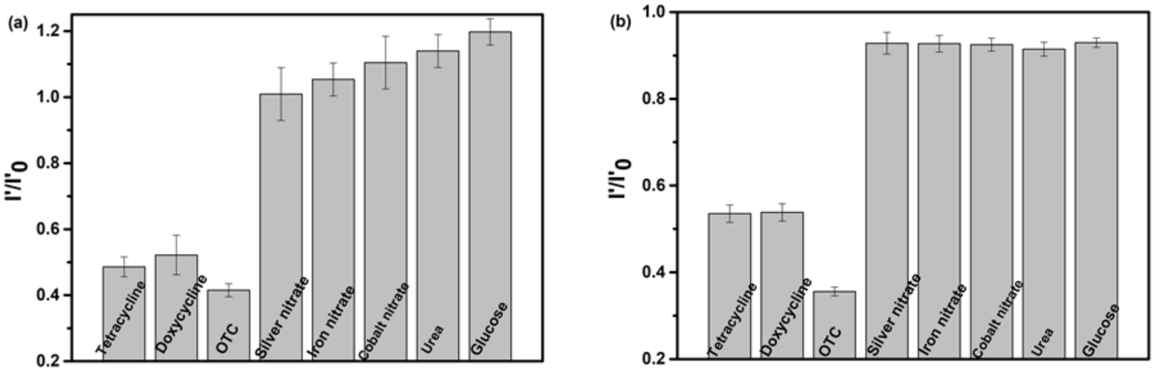

Figure S6. Selectivity of N-CQDs: (**a**) ON-CQDs; (**b**): WN-CQDs. Concentrations of various substances: (**a**), (**b**) correspond to 40 μmol L^{-1} and 80 μmol L^{-1} respectively. (I′ and I'_0 represent the fluorescence intensity of N-CQDs in the presence and absence of various substances, respectively.).

Table 1. Compare OTC recovery rates in various samples.

Samples	Spiked	Recovery Rate (%)	RSD (%, n = 5)	Citations
River water	5 μmol L^{-1}	98.73	1.26 %	
	15 μmol L^{-1}	104.28	1.22%	[1]
	25 μmol L^{-1}	103.42	1.32%	
	1 μmol L^{-1}	104	0.11%	
Milk	5 μmol L^{-1}	101	0.21%	[3]
	10 μmol L^{-1}	99	0.24%	
	10 μmol L^{-1}	107.9	1.49%	
Honey	20 μmol L^{-1}	107.2	1.46%	[4]
	30 μmol L^{-1}	101.9	0.75%	
Pork	10 μmol L^{-1}	109.5	1.74%	
	20 μmol L^{-1}	98.3	1.19%	[4]
	30μmol L^{-1}	104.9	1.09%	
Tap Water	10 μmol L^{-1}	97.5	0.94%	
Soil	12.240μmol Kg^{-1}	103.2	0.82%	This work
Lake Water	40 μmol L^{-1}	100.9	0.62%	

References

1. Qi, H.J.; Teng, M.; Liu, M.; Liu, S.X.; Li, J.; Yu, H.P.; Teng, C.B.; Huang, Z.H.; Liu, H.; Shao, Q., et al. Biomass-derived nitrogen-doped carbon quantum dots: highly selective fluorescent probe for detecting Fe^{3+} ions and tetracyclines. *J. Colloid. Interf. Sci.* **2019**, *539*, 332-341.
2. Xu, Z.Q.; Yi, X.F.; Wu, Q.; Zhu, Y.C.; Ou, M.R.; Xu, X.P. First report on a Bodipy-based fluorescent probe for sensitive detection of oxytetracycline: application for the rapid determination of oxytetracycline in milk, honey and pork. *RSC Adv.* **2016**, *6*, 89288-89297.
3. Xu, N.; Yuan, Y.Q.; Yin, J.H.; Wang, X.; Meng, L. One-pot hydrothermal synthesis of luminescent silicon-based nanoparticles for highly specific detection of oxytetracycline *via* ratiometric fluorescent strategy. *RSC Adv.* **2017**, *7*, 48429-48436.
4. Chandra, S.; Ghosh, B.; Beaune, G.; Nagarajan, U.; Yasui, T.; Nakamura, J.; Tsuruoka, T.; Baba, Y.; Shirahata, N.; Winnik, F.M. Functional double-shelled silicon nanocrystals for two-photon fluorescence cell imaging: spectral evolution and tuning. *Nanoscale* **2016**, *8*, 9009-9019.
